# Pharmacokinetics and cardiac safety of clofazimine in children with rifampicin-resistant tuberculosis

**DOI:** 10.1128/aac.00794-23

**Published:** 2023-12-19

**Authors:** Ali Mohamed Ali, Belén P. Solans, Anneke C. Hesseling, Jana Winckler, H. Simon Schaaf, Heather R. Draper, Louvina van der Laan, Jennifer Hughes, Barend Fourie, James Nielsen, Lubbe Wiesner, Anthony J. Garcia-Prats, Radojka M. Savic

**Affiliations:** 1 Department of Bioengineering and Therapeutic Sciences, University of California San Francisco, San Francisco, California, USA; 2 Department of Interventions and Clinical Trials, Bagamoyo Research and Training Center, Ifakara Health Institute, Bagamoyo, Tanzania; 3 Department of Paediatrics and Child Health, Desmond Tutu TB Centre, Faculty of Medicine and Health Sciences, Stellenbosch University, Cape Town, South Africa; 4 Department of Pediatrics, New York University School of Medicine, New York, New York, USA; 5 Department of Medicine, Division of Clinical Pharmacology, University of Cape Town, Cape Town, South Africa; 6 Department of Pediatrics, University of Wisconsin-Madison, School of Medicine and Public Health, Madison, Wisconsin, USA; Bill & Melinda Gates Medical Research Institute, Cambridge, Massachusetts, USA

**Keywords:** pharmacokinetics, tuberculosis, clofazimine, pediatrics, QT prolongation

## Abstract

Clofazimine is recommended for the treatment of rifampicin-resistant tuberculosis (RR-TB), but there is currently no verified dosing guideline for its use in children. There is only limited safety and no pharmacokinetic (PK) data available for children. We aimed to characterize clofazimine PK and its relationship with QT-interval prolongation in children. An observational cohort study of South African children <18 years old routinely treated for RR-TB with a clofazimine-containing regimen was analyzed. Clofazimine 100 mg gelatin capsules were given orally once daily (≥20 kg body weight), every second day (10 to <20 kg), or thrice weekly (<10 kg). PK sampling and electrocardiograms were completed pre-dose and at 1, 4, and 10 hours post-dose, and the population PK and Fridericia-corrected QT (QTcF) interval prolongation were characterized. Fifty-four children contributed both PK and QTcF data, with a median age (2.5th–97.5th centiles) of 3.3 (0.5–15.6) years; five children were living with HIV. Weekly area under the time-concentration curve at steady state was 79.1 (15.0–271) mg.h/L compared to an adult target of 60.9 (56.0–66.6) mg.h/L. Children living with HIV had four times higher clearance compared to those without. No child had a QTcF ≥500 ms. A linear concentration-QTcF relationship was found, with a drug effect of 0.05 (0.027, 0.075) ms/µg/L. In some of the first PK data in children, we found clofazimine exposure using an off-label dosing strategy was higher in children versus adults. Clofazimine concentrations were associated with an increase in QTcF, but severe prolongation was not observed. More data are required to inform dosing strategies in children.

## INTRODUCTION

Rifampicin-resistant tuberculosis (RR-TB), including multidrug-resistant (MDR) tuberculosis (TB), presents a major threat to the fight against TB worldwide. According to the World Health Organization (WHO), in 2019, there were an estimated 465,000 (400,000–535,000) new cases of RR/MDR-TB globally (1). Mathematical modeling studies suggest that 25,000–32,000 new MDR-TB cases occur in children annually (2). Treatment of MDR/RR-TB requires the use of second-line drugs in longer regimens than those used for drug-susceptible (DS)-TB. In 2022, the WHO issued new guidance for older adolescents and adults for the use of the 6-month all-oral regimens bedaquiline-pretomanid-linezolid-moxifloxacin and bedaquiline-pretomanid-linezolid as priority treatment for RR/MDR-TB for eligible older adolescents (≥14 years) and adults. Clofazimine may play less of a role in the short term for adolescents and adults with RR/MDR-TB. However, because of delays in pediatric development, children will not be able to access pretomanid for some time. Therefore, the WHO still recommends clofazimine as part of standard 9–11 month regimens for RR/MDR-TB treatment in children or as a group B medicine to be used for RR/MDR-TB patients on individually constructed longer treatment regimens (3, 4).

Clofazimine is a highly lipophilic compound that accumulates in fatty tissues (5 – 7), has a large volume of distribution (8), is metabolized in the liver with minimal renal excretion, and has a half-life of 10–70 days in adults (5, 9, 10). Both bioavailability and absorption rate constants are enhanced when taken with food (9, 11). Clofazimine demonstrated concentration-dependent antimicrobial activity in adults with pulmonary TB (12). In adults with MDR-TB, clofazimine-containing regimens were effective (more than 80% cure achieved) and contributed to decreasing the required duration of therapy from 18 to 24 months to 9–12 months (5, 13, 14). While several studies have reported the safety and pharmacokinetics (PK) of clofazimine in adults (8, 11), data in children are lacking.

The main safety concern for clofazimine is QT-interval prolongation (15 – 17). Several studies in adults have confirmed the QT-prolonging effects of clofazimine (18 – 20). This effect increased when clofazimine was used in combination with other QT-prolonging drugs such as bedaquiline and moxifloxacin (21, 22). In a study of adults with DS-TB receiving clofazimine, clofazimine had a significant QT prolongation effect driven by its concentration (23).

Currently, there are no verified dosing guidelines for the use of clofazimine in children due to a lack of PK and safety data in children. Characterizing PK and safety is crucial to supporting safe and effective use of clofazimine in children. Our goal was to characterize and report clofazimine PK and QT interval prolongation data in a cohort of children 0 to <18 years of age routinely treated for RR/MDR-TB.

## MATERIALS AND METHODS

### Study design, patients, and treatment

A prospective, observational cohort study in South Africa of PK and safety of key second-line TB drugs in children (<18 years) was conducted. Detailed information on study design and methods has been previously described (24). Children routinely treated for RR-TB from 2016 to 2020 were enrolled, irrespective of HIV status. Ideally, children ≥8 years of age would have routinely received moxifloxacin, and children <8 years of age would receive levofloxacin in addition to at least three other effective TB drugs for 9–18 months (25, 26). The PK sampling approach was designed to accommodate the PK profile of multiple TB drugs. Clofazimine was administered as part of multi-drug regimens for RR-TB consisting, among others, of bedaquiline, delamanid, and moxifloxacin.

Children received 100 mg soft gelatin capsules (gel capsules) of clofazimine at a target daily dose of approximately 2–5 mg/kg. The drug was given orally on an empty stomach after an overnight fast with water, once daily for children weighing more than 20 kg, every second day for 10–20 kg children, or thrice weekly for children weighing less than 10 kg. A meal was given to each patient 1 hour after dosing. For children unable to swallow the capsules whole, the capsules were placed in a small amount of yogurt; the capsules did not completely dissolve but softened substantially so they could be opened or more easily administered to young children, and then administered to the child who could swallow or chew them. All children living with HIV received antiretroviral treatment (ART) at study enrollment as per South African treatment guidelines.

### Pharmacokinetic sampling and analysis

A semi-intensive PK sampling strategy was used, following at least 2 weeks after treatment initiation. An opportunistic sampling design was used, and blood samples were collected pre-dose and at 1, 4, and 10 hours after drug administration. A subset of study participants had PK sampling on two occasions while taking two different formulations of either levofloxacin or moxifloxacin; therefore, samples were collected on two or more PK days.

Clofazimine concentration was analyzed with a validated liquid chromatography tandem mass spectrometry assay developed at the Division of Clinical Pharmacology, University of Cape Town (see Supplemental Materials).

Clofazimine concentration-time measurements were modeled using non-linear mixed effects. Population PK parameter estimates were obtained using the first-order conditional estimation method with interaction. Between-individual and between-occasion variability (BOV) in the PK parameters was modeled exponentially. One patient had unscheduled visits (visits 4 and 5) but no dose was given during these visits therefore the PK data for these visits were not used. One- and two-compartment disposition models with first-order elimination were investigated. First-order absorption models with and without lag time and transit compartment absorption were tested (27). Allometric scaling by body weight, fat-free mass and total fat was tested in both clearance and volume of distribution. Fat-free mass (FFM) was calculated using the empirical model (28) as follows:

$FFM=\left(\alpha +\frac{\left(1-\alpha \right)}{1+{\left(\frac{AGE}{{AGE}_{50}}\right)}^{-\gamma }}\right)\times \left(\frac{9270\times WT}{\beta +\left(\theta \times BMI\right)}\right),$

where α is the lower bound of the sigmoid hyperbolic function and has values of 0.88 and 1.11 for males and females, respectively; γ is the sigmoidicity coefficient and has values of 12.7 and 1.1 for males and females, respectively; and AGE_50_ is the FFM maturation half-life and has values of 13.4 and 7.1 for males and females, respectively. “WT” denotes body weight in kilograms, “BMI” denotes body mass index in kilograms/meter^2^, β have values of 6680 and 8780 for males and females, respectively, and θ have values of 216 and 244 for males and females, respectively. Fatmass was calculated by subtracting FFM from body weight (Fatmass = WT – FFM). Model building was primarily guided by improvements in the objective function value, goodness-of-fit plots, and visual predictive checks.

To assess the influence of covariates on the PK characteristics of clofazimine, a stepwise covariate modeling approach with forward inclusion of *P* < 0.05 and backward elimination of *P* < 0.01 was used (29). Covariates tested for inclusion were age, sex, formulation (whole capsules vs capsules mixed in yogurt/opened), ethnicity, HIV status, concomitant medications, and nutritional status. A child was considered undernourished if they had a weight for age z-score (WAZ) <−2 in children <10 years or a body mass index for age z-score (BAZ) <−2 in children ≥10 years. For children older than 10 years, BAZ was used to define underweight instead of WAZ since WAZ is inadequate in this age group due to its inability to distinguish between relative height and body mass (30). WAZ and BAZ were calculated based on WHO-defined nutrition metrics (31, 32). The selection of covariates was informed by statistical and clinical significance and physiological plausibility.

### QT interval prolongation and safety assessment

A 12-lead electrocardiogram (ECG) was performed in triplicate at pre-dose and at 1, 4, and 10 hours after drug administration on the day of PK sampling. The QT intervals were corrected for the effect of heart rate using the Fridericia formula (33). The mean of the triplicate QT intervals was used for descriptive analysis, whereas all observations were used for modeling.

The Fridericia-corrected QT interval (QTcF) data were modeled in Nonlinear Mixed Effect Mode (NONMEM), where both the PK parameter estimates and PK data were used to estimate the QTcF model parameters. Children who had both ECG and PK measurements were used to characterize the clofazimine concentration-QTcF relationship. Both linear and E_max_ (the maximum effect a drug can have) models were evaluated. Between-individual and between-occasion variability was modeled exponentially. A stepwise covariate modeling procedure as described above was used to test the effect of age, nutritional status, HIV status, time on clofazimine treatment, and use of concomitant QT-prolonging agents on baseline (pre-dose) and drug-effect parameters (slope of the linear model).

### Simulations

The PK model developed by Abdelwahab et al. (8) was used to simulate target concentration in adults. We generated *in silico* data for a 48- to 61-kg adult who received a loading dose of 300 mg daily on days 1–3, followed by 100 mg daily on days 4–14, and simulated (*N* = 1,000) clofazimine concentrations over time for 6 months and compared them with observed clofazimine concentrations in children. The final model developed with children’s data was used to derive a weekly area under the time-concentration curve (AUC) at steady state and to compare it with a calculated weekly target exposure in adults (8, 34). Weekly AUC was used to normalize results between children who received different dosing regimens.

### Statistics and software

NONMEM 7.4 and Perl-speaks-NONMEM 4.7.0 (Icon Development Solutions, Ellicott City, MD) were used for modeling and simulation. STATA (version 15; Stata Corp., College Station, TX, USA) and R Statistical Software (version 3.4.3, https://www.r-project.org/) were used for descriptive and graphical analysis. A drop in objective function value greater than 3.84, 7.88, and 10.83 was considered significant at the 5, 0.5, and 0.1% levels, respectively, for nested models differing in one parameter. For non-nested models, the Akaike Information Criteria (AIC) was used. The *t*-test (normal) or Wilcoxon rank-sum test (non-normal) was used to test the differences in baseline characteristics for continuous variables, while the Chi-squared or Fisher Exact test (when the expected cell frequency was <5 in more than 20% of the cells) was used for categorical variables. Visual diagnostics were done with “Xpose” (0.4.4) and “vpc” (1.0.1) R packages. The precision of the final parameter estimates was evaluated using a non-parametric bootstrap with replacement (*n* = 1,000) done in NONMEM.

## RESULTS

### Patients and sampling

**Fig 1:**
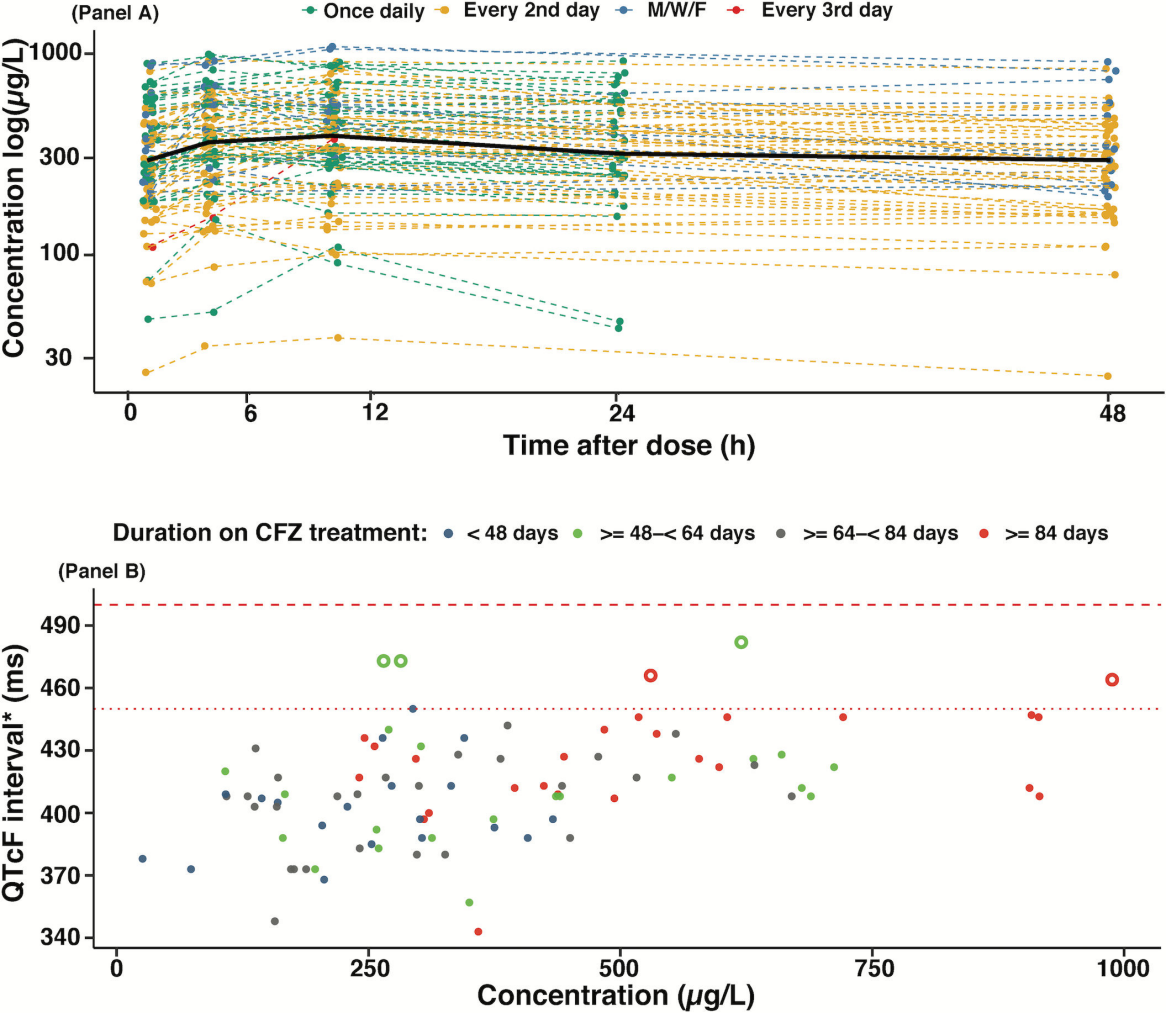
Clofazimine drug concentration by dosing frequency (panel A) and maximum QTcF by clofazimine concentration (panel B). Solid black line in panel A is the median clofazimine concentration. Panel B: *each solid circle represents a maximum QTcF at each pharmacokinetic visit. Solid circles are maximum QTcF values less than or equal to 450 ms. Open circles have maximum QTcF values greater than 450 ms. The dotted red line represents a QTcF of 450 ms, and the dashed red line represents a QTcF of 500 ms (grade three adverse event). QTcF, QT interval corrected by the Fridericia formula; M/W/F, Monday/Wednesday/Friday.

The 54 participants’ baseline characteristics with both PK and ECG measurements are presented in Table 1. Thirty-six (67%) children were under 5 years of age. Eleven (20%) children received other QT-prolonging TB drugs (bedaquiline, delamanid, and/or moxifloxacin), 6 of which (55%) received moxifloxacin. Five (9.3%) children were living with HIV; the use of clofazimine with antiretroviral (ARV) regimens in these children is presented in Table 1. Out of the 6 undernourished children, 2 (33%) were ≥5 years of age. The median (2.5th–97.5th) weekly clofazimine dose was 31 (14–48) mg/kg. The use of clofazimine with other QT-prolonging drugs was highly associated with age (Fisher’s exact test, *P* = 0.001), where 33 (94.3%) of children ≥5 years received clofazimine with other QT-prolonging drugs compared to 10 (52.6%) of children <5 years. The association between drug use and the patient’s characteristics is presented in Table S1. The 54 children contributed 370 PK samples (Fig. 1A). Thirty-four (63%) and five (9%) children had two and three sampling occasions, respectively (Table S2). The median (2.5th–97.5th centiles) maximum concentration (C_max_) for clofazimine was 0.49 (0.146–0.965) mg/L. No samples were below the lower limit of quantification (0.00781 mg/L).

**TABLE 1:** Baseline characteristics of clofazimine pharmacokinetics and safety study participants^*b*^ ^,*c*^

Variable	Value
Number of children	54
Patient information	
Female, *n* (%)	29 (54)
Age (year); median (2.5th–97.5th centiles) in years	3.3 (0.5, 15.6)
<5 years, *n* (%)	36 (67)
Weight; median (2.5th–97.5th centiles) in kg	13.3 (6.7, 51.2)
HIV-positive, *n* (%)	5 (9)
ART regimen (of five children living with HIV)	
Abacavir-Lamivudine-Efavirenz	2/5
Abacavir-Lamivudine-Lopinavir/Ritonavir	1/5
Abacavir-Lamivudine-Nevirapine	1/5
Emtricitabine-Tenofovir-Lopinavir/Ritonavir	1/5
Formulation of clofazimine	
Whole capsule, *n* (%)	48 (89)
Opened capsules, *n* (%)	6 (11)
Height; median (2.5th–97.5th centiles) in cm	94 (65, 164)
BMI; median (2.5th–97.5th centiles) in kg/m^2^	15.8 (13.0, 20.6)
BAZ; median (2.5th–97.5th centiles)	−0.1 (−2.8, 1.5)
WAZ^*a*^; median (2.5th–97.5th centiles)	−0.58 (−3.2, 1.4)
HAZ^*a*^; median (2.5th–97.5th centiles)	−1.1 (−3.1, 1.6)
Undernourished, *n* (%)	6 (11)
Dosing information	
Weekly clofazimine dose; median (2.5th–97.5th centiles) in (mg/kg)	31 (14, 48)
Dosing frequency, *n* (%)	
Once daily	19 (35)
Every second day	25 (46)
Monday/Wednesday/Friday	9 (17)
Every third day	1 (2)
Concomitant QT prolongation medication, *n* (%)	
Bedaquiline	3 (6)
Delamanid	1 (2)
Moxifloxacin	6 (11)
Bedaquiline + Delamanid	1 (2)

^*a*^ Children <5 years only.

^*b*^ Obtained from weight for age z-score for children <5 years (*n* = 36) and body mass index for age z-score for children ≥5 years (*n =* 28)*.*

^*c*^ BMI: body mass index; BAZ: body mass index for age z-score; HAZ: height for age z-score; WAZ: weight for age z-score.

### Population pharmacokinetics

The population PK of clofazimine was best described as a one-compartment distribution with first-order absorption and elimination. The introduction of allometric scaling by body weight on clearance and volume of distribution resulted in a better fit (AIC = 3,749) compared to fat and fat-free mass (AIC = 3,809). HIV was identified as a significant covariate on clearance (CL/F), with children living with HIV having four times higher CL/F compared to children living without HIV. Age significantly affected the volume of distribution (Vd/F), with a 32% increase in Vd/F per year of age. No other covariates had an effect on PK characteristics. The final PK parameter estimates are presented in Table 2. Prediction-corrected visual predictive checks indicated that the model predicted the observed data well (Fig. S1).

**TABLE 2:** Population pharmacokinetic and QTcF parameter estimates^*h*^

Parameter	Value (% RSE), [95% CI]	IIV/BOV^*g*^ %CV ^*e*^ (% RSE), [95% CI]
Pharmacokinetic model		
CL/F^*a*^ (L/h)	4.74 (20.4), [3.07, 6.46]	77.7% (20.1), [17.2, 117]
Vd/F^*b*^ (L)	3200 (22.4), [1970, 5310]	
Ka (1/h)	1.02 (66.6), [0.358, 4.47]	
Residual error, proportional (%)	16.4 (18.4), [10.3, 20.6]	
Residual error, additive (µg/L)	24.6 (57.7), [0.246, 43.4]	
Bioavailability^*c*^	1^*f*^	38.9% (18.5), [24.4, 68.7]^*g*^
Covariate effect		
Effect of age on bioavailability	0.19 (27.7), [0.057, 0.26]	
Effect of age on Vd/F	0.318 (4.8), [0.071, 0.337]	
Effect of HIV on CL/F	2.91 (68.4), [0.51, 4.98]	
QTcF model ^*d*^		
Baseline parameter (ms)	360 (1.4), [350, 370]	15.1 (13.2), [10.4, 18.3]
Slope (ms/µg/L)	0.050 (26.0), [0.027, 0.075]	0.028 (22.6), [0.015, 0.043]^*g*^
Residual error, additive (ms)	15.3 (5.4), [13.7, 16.9]	
Covariate effect		
Effect of age on baseline parameters	0.009 (17.3), [0.006, 0.012]	

^*a*^ CL/F = θ_pop_ ⋅ (WT/13.3)3/4.(1 + θ_HIV_).

^*b*^ Vd/F = θ_pop_ × (WT/13.3)^1^.[1 + θ_age_ × (age – 3.35 years)].

^*c*^ Bioavailability = θ_pop_ × [1 + θ_age_ × (age – 3.35 years)].

^*d*^ QTcF = Baseline × [1 + θ_age_ × (age – 3.35 years)] + Slope × C_p_ + e.

^*e*^ Inter-individual variability was modeled exponentially for pharmacokinetic parameters and additively for QTcF parameters.

^*f*^ Bioavailability is fixed to 1.

^*g*^ Between occasion variability (intra-individual variability).

^*h*^ CL/F: apparent clearance; Vd/F: apparent volume of distribution; Ka: absorption rate constant; θ_pop_ : population estimate; WT: individual body weight; CL/F and Vd/F refer to a patient weighing 13.3 kg, the median weight in the data set; θ_HIV_ : effect of HIV positive status on CL/F; θ_age_: effect of age on Vd/F and baseline QTcF, centered at the population median of 3.35 years; C_p_: concentration of clofazimine in plasma; QTcF: QT-interval corrected by Fridericia formula; RSE: relative standard error; 95% CI: 95% confidence interval based on non-parametric bootstrap (*n* = 1,000).

### Cardiac safety

**Fig 2:**
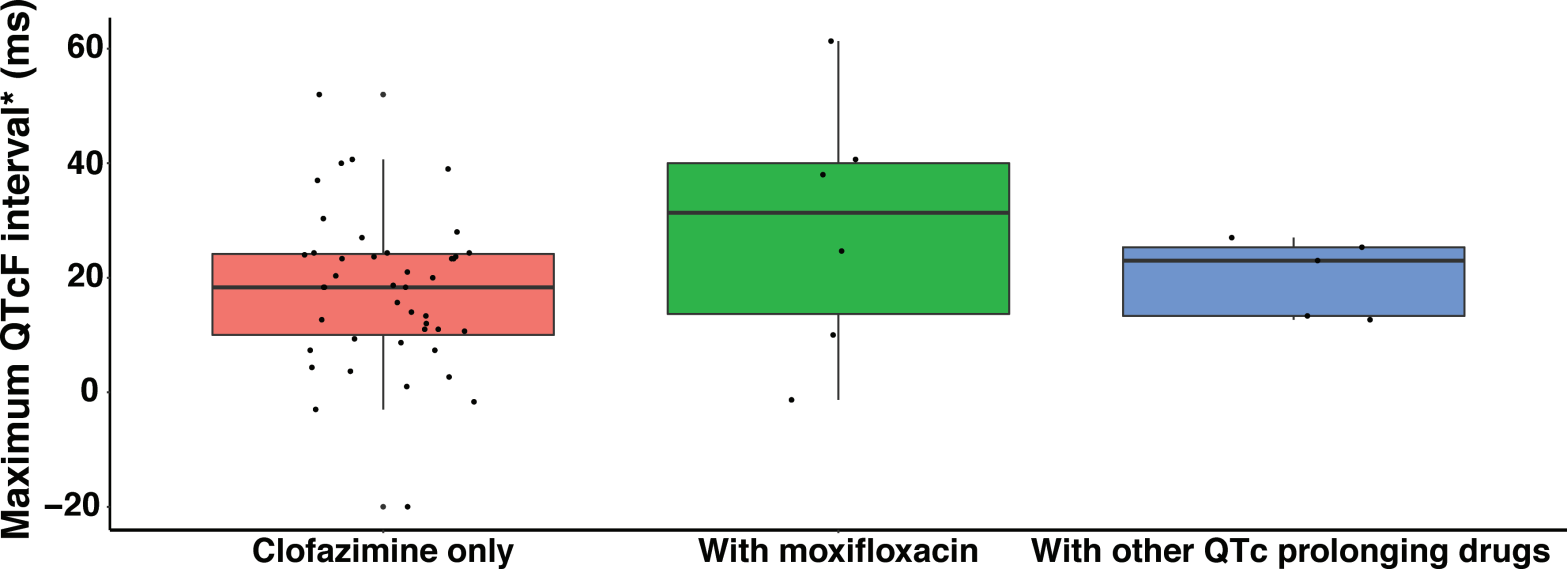
Maximum change in QTcF during the dosing interval in children receiving only clofazimine (*n* = 43) or clofazimine with other QTc-prolonging drugs (*n* = 11). Boxplots represent the median and interquartile range, and whiskers show the 95th and 5th percentiles, respectively. *Each solid circle represents a maximum change in QTcF during clofazimine treatment. QTcF = Fridericia-corrected QT interval.

Fifty-four children contributed 1,156 ECG measurements after repeated oral dosing of clofazimine (patients received clofazimine for >3 months). Thirty-nine (72%) and 6 (11%) of these 54 children contributed ECG data on two and three occasions, respectively. The number of children who had ECG measurements at the time of PK sampling is shown in Table S2. Median (2.5th–97.5th centiles) pre-dose QTcF was 385 (331–439) ms, and the maximum QTcF was 412 (362–470) ms, reached at 1.3 (0–10.4) hours after the dose, but no child had a QTcF interval >500 ms during the PK sampling occasions. Median (2.5th–97.5th centiles) duration of treatment with clofazimine up to the maximum observed QTcF was 64 (10–162) days (Table 3). There was an increasing QTcF the longer the child was on treatment (Fig. S2). There were 4 (4%) out of 98 occasions with QTcF >450 to ≤480 ms; 1 of 4 occasions with QTcF >450 ms occurred in a child receiving moxifloxacin. The maximum change in QTcF was higher (31.3 ms) in children who received clofazimine with moxifloxacin compared to 18.3 ms in children who received only clofazimine and 23.0 ms in children who received other QTcF-prolonging drugs (Fig. 2). However, this difference was not statistically significant (Kruskal-Wallis test, *P* = 0.3483). The maximum increase in the QTcF from the pre-dose measure over the PK sampling timeframe was 18.3 ms and 24.7 ms (Wilcoxon-Mann-Whitney test, *P* = 0.1659) in children treated with clofazimine without and with other QT-prolonging drugs, respectively.

**TABLE 3:** Clofazimine effect on QTcF-interval on pharmacokinetic sampling day

Variable	Value
Number of children^*a*^	54
Pre-dose QTcF; median (2.5th–97th centiles) in ms	385 (331, 439)
QT prolongation events	4
Grade 1 (mild)	4
Grade 2 (moderate)	0
Grade 3 (severe)	0
Maximum △QTcF pre-dose to max post-dose; median (2.5th–97th centiles) in ms	12 (−23, 41)
Events with △QTcF >30 ms <60 ms	8
Events with △QTcF ≥60 ms	1
Maximum QTcF; median (2.5th–97th centiles) in ms	412 (362, 470)
Time after dose at maximum QTcF; median (2.5th–97th centiles) in hours	1.3 (0, 10.4)
Days on clofazimine before PK day; median (2.5th–97.5th centiles) in days	64 (10, 162)

^*a*^ A patient can appear multiple times due to multiple PK visit. QTcF: QT interval corrected by Fridericia formula; DQTcF: change in QTcF from time 0 over PK sampling interval; mild QT prolongation: QTcF interval >450 to ≤480 ms; moderate QT prolongation: QTcF interval >480 to ≤500 ms; severe QT prolongation: QTcF interval >500 ms.

### Clofazimine concentration-QTcF relationship

**Fig 3:**
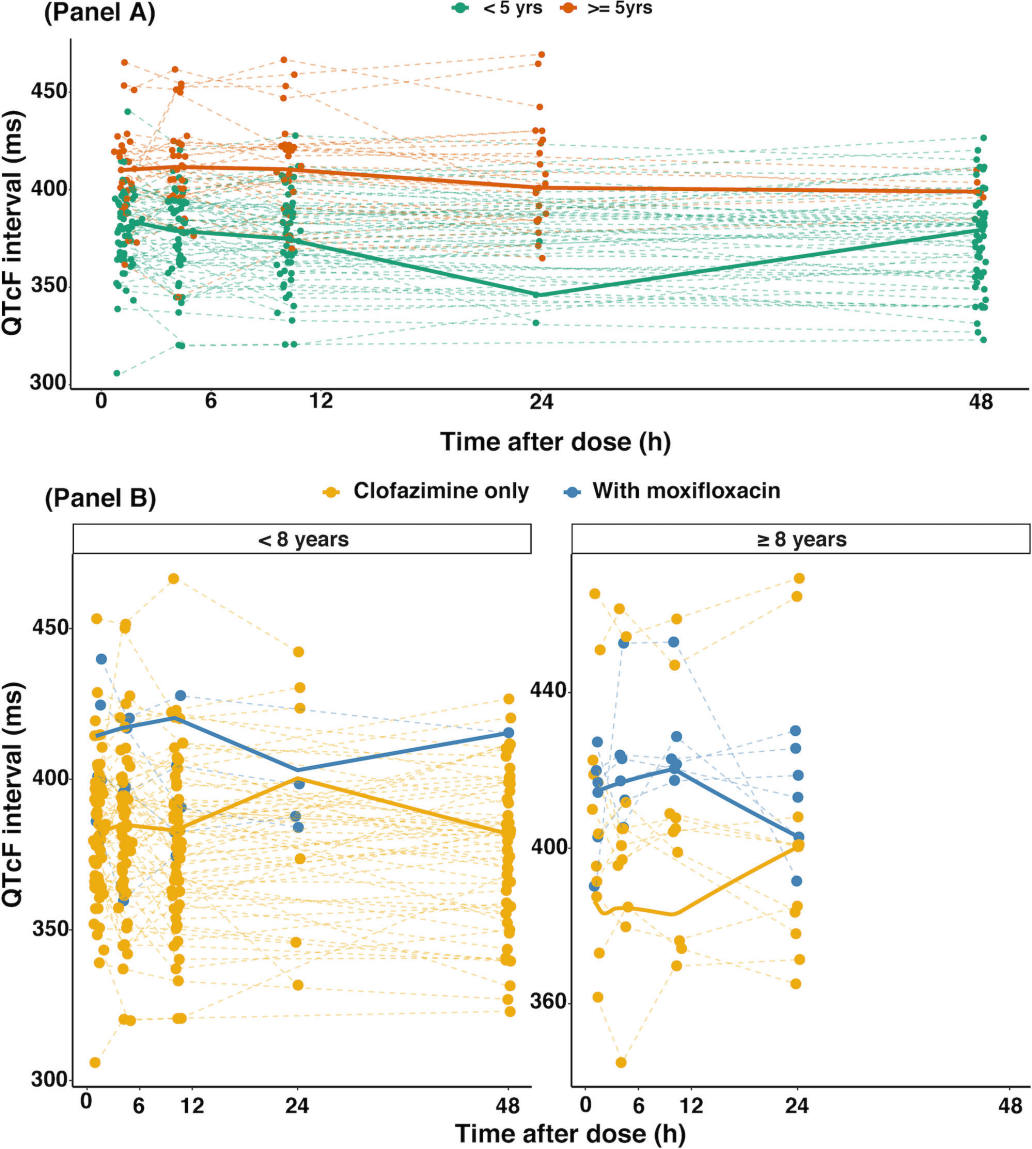
QTcF profiles in children treated with clofazimine for rifampicin-resistant tuberculosis. Dashed lines represent distinct children and sampling occasions, and dots represent individual observations. QTcF = Fridericia-corrected QT interval. The bold lines are the population median. Panel A: QTcF profiles in children treated with clofazimine for rifampicin-resistant tuberculosis by age group. Panel B: QTcF profiles in children treated with clofazimine for rifampicin-resistant tuberculosis stratified by the use of moxifloxacin.

Clofazimine-induced QTcF prolongation was best characterized by a direct relationship using a linear model (Fig. 1B). For each unit (µg/L) of increase in clofazimine concentration, there was an estimated 5.0% increase in QTc prolongation. An E_max_ model had a similar fit as the linear model, but the relative standard error was very high for both Emax (3274%) and C_50_ (3775%). When fixing the value of C_50_ from the value obtained in adults (23), the E_max_ model had an AIC value of 12,600 compared to the value of 12,593 of the linear model, and therefore the E_max_ was not further considered. The PK-QTcF model estimates are shown in Table 2. Age was the only significant covariate affecting pre-dose QTcF; there was a 3 ms increase in pre-dose QTcF per year of age (Fig. 3A). The use of moxifloxacin in addition to clofazimine increased pre-dose QTcF by 6% (Fig. 3B), but this effect was not statistically significant after controlling for age. The time on clofazimine treatment was not statistically significant. The final model predicted the QTcF interval data well (Fig. S2).

### Simulations

**Fig 4:**
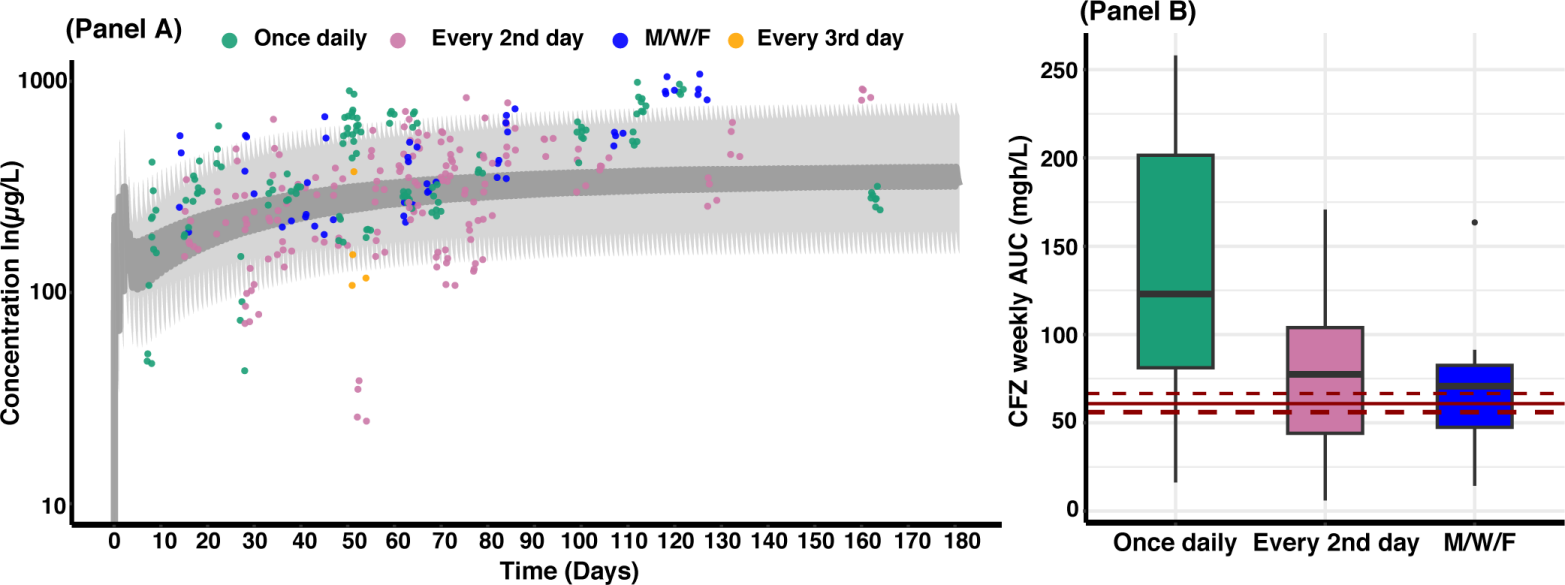
Pharmacokinetic profile for children and simulated adult profile for 6 months of treatment (Panel A) and clofazimine weekly area under the curve (AUC) at steady state (Panel B). Panel A: solid circles represent child-observed concentrations over time at unique sampling occasions, stratified by dosing regimen. Gray solid line represents the simulated adult median concentration. Shaded area is the 95% CI of the simulated adult concentration. Panel B: solid and dashed red lines are the median and the 25th and 75th percentiles of adult weekly AUC, respectively.

Simulated adult clofazimine concentrations along with the measured clofazimine concentrations from children in this study are presented in Fig. 4A. Clofazimine exposures were generally comparable between children of different weights; however, younger children (≤20 kg) who received clofazimine every second day or three times weekly had lower exposure values compared to older children (>20 kg) who received clofazimine once daily (Fig. 4B). Weekly AUC at steady state was 85.3 (15.0–410.0) mg.h/L and 60.9 (56.0–66.6) mg.h/L for children and adults (7), respectively.

## DISCUSSION

This is the first report, to our knowledge, of the pharmacokinetics and cardiac safety of clofazimine in young children with RR/MDR-TB. Our model predicted an overall higher weekly AUC at steady state compared to an adult model (8), and this is expected because normally AUC estimates are more variable in children than in adults. Concentration-QTcF relationship was best explained with a linear model. We found an effect of HIV infection and age on CL/F and Vd/F, respectively. None of the children experienced QTcF >500 ms receiving current dosing (2–7 mg/kg/day) of clofazimine.

Our study found four times higher clearance in children living with HIV compared to children not living with HIV. However, only five children in our study population were living with HIV, and this finding needs to be interpreted with caution. In a study of adults in South Africa, HIV was not found to affect clofazimine PK. However, there was an effect of lopinavir/ritonavir on clofazimine bioavailability in adults, leading to higher clofazimine exposure, but the effect was not statistically significant (8). Clofazimine has been reported to inhibit the major metabolic pathways of ARVs, including CYP3A4, CYP2B6, CYP2C9, and CYP219, and hence can be thought of as causing significant CYP-enzyme-mediated drug–drug interactions with ARVs (35). Clofazimine has been used in adults living with HIV (36 – 38), and it is not clear whether the effect we found in this study was due to HIV co-infection itself or due to drug–drug interactions that may have affected clofazimine PK characteristics. Quantification of the effect of HIV and drug–drug interactions merits further evaluation.

In our study, we found Vd/F increased with age. Nonpolar compounds are normally lipid-soluble, and therefore their Vd/F increase with age (39), as with age, body fat increases and total body water and lean body mass decrease (40). Clofazimine is a highly lipophilic compound (5 – 7), and hence we expected it to have larger volumes of distribution as the child gets older (41) or when body total fat mass increases; however, the fat mass and fat-free mass performed worse on an allometric scale than total body weight.

Clofazimine has been associated with QTc interval prolongation in adults (18 – 20, 23). The concentration-QTc relationship in children has not previously been described. We found an association between clofazimine and QTcF prolongation, with a linear drug-effect model describing the relationship well. Abdelwahab and colleagues (23) found an E_max_ drug-effect model best describing the clofazimine concentration-QTc relationship in adults with TB, but our data did not support the use of an E_max_ model over a linear one. Our concentration-QTc model should not be used for extrapolation outside the range of the data used in this analysis. More data are needed to establish a more physiological concentration-QTc model (E_max_).

The use of clofazimine and moxifloxacin together has been associated with increased QTc prolongation in adults (22, 42). In our study, only six children received both clofazimine and moxifloxacin, and hence we did not have enough power to detect the effect of the combination on QTcF prolongation. Ali and colleagues (43) found that the risk of QTcF interval prolongation is highest (a 1.4-fold increase in QTcF) in children when clofazimine and moxifloxacin are used together. In an adult study of Korean patients, QTcF interval prolongation was found to occur at a greater magnitude in patients receiving clofazimine and moxifloxacin together compared to those receiving clofazimine or moxifloxacin as a single drug (42). Radtke et al. (22) found that the use of clofazimine and moxifloxacin together increased E_max_ from 8.8 ms to 28 ms but did not increase pre-dose QTcF. In our study, we observed a 6% increase (from 360 ms to 382 ms) in pre-dose QTcF in patients receiving clofazimine and moxifloxacin together. However, this effect was not significant when age was included in the pre-dose QTcF, probably because older children (≥5 years) were administered clofazimine and moxifloxacin more than younger children (<5 years). Given clofazimine’s long elimination half-life (10–70 days) in adults and delayed activity (5, 9, 10, 12, 44, 45), it is possible that it prolongs the QTc interval over a longer duration; however, the impact of treatment duration on the magnitude of QTc prolongation was not statistically significant in our study. ECGs should be monitored longitudinally in children on clofazimine and other QT-prolonging agents.

We found that children >20 kg had slightly higher clofazimine exposure compared to children ≤20 kg; however, we did not attempt to simulate alternative dosing strategies because of the study limitations. More data from additional trials are needed to guide the optimal dosing of clofazimine in children with RR/MDR-TB.

Limitations of our study include the opportunistic sampling design for clofazimine and the absence of a true “baseline” QTcF (i.e., prior to the initiation of treatment). While food increases the bioavailability of clofazimine (11), in this study, clofazimine was given on an empty stomach, and no data on the food they received 1 hour after they received the medication were available. As a result, the effect of food on clofazimine PK parameters could not be quantified. A pre-dose QTcF measure was used instead of a true baseline measure. Consequently, the effect of the drug on QTcF prolongation could have been underestimated by our model.

In conclusion, this is the first study reporting PK properties and concentration-QTc relationship of clofazimine in children with RR-TB. Our results show that clofazimine, with the dosing strategy used in this study, was safe and was not associated with severe QTcF prolongation in children with RR-TB. However, more data are required on the safety of clofazimine, especially when used in combination with other QT-prolonging drugs. The higher clearance found in children with HIV needs further evaluation because our study included a limited number of children living with HIV.

Future PK studies on clofazimine in children should utilize more optimal design and sampling strategies for the quantification of developmental pharmacology.

## SUPPLEMENTAL MATERIAL

Supplemental material aac.00794-23-s0001.pdf Supplemental methods, Tables S1 and S2, and Fig. S1 to S3.

ASM does not own the copyrights to Supplemental Material that may be linked to, or accessed through, an article. The authors have granted ASM a non-exclusive, world-wide license to publish the Supplemental Material files. Please contact the corresponding author directly for reuse.
