# Supplementary material for: Pharmacokinetics and cardiac safety of clofazimine in children with rifampicin-resistant tuberculosis
Source: Antimicrob Agents Chemother. 2023 Dec 19;68(1):e00794-23. doi: 10.1128/aac.00794-23 (PMC10777824; doi:10.1128/aac.00794-23)
Supplement: Supplemental material — Supplemental methods, Tables S1 and S2, and Fig. S1 to S3. [file aac.00794-23-s0001.pdf]

Supplementary

Method

Pharmacokinetic sampling and analysis

The plasma assay consisted of a protein precipitation extraction, followed by high performance liquid chromatography with tandem mass spectrometry detection. The extraction procedure was followed by isocratic liquid chromatographic separation using a Phenomenex Synergi Hydro-RP (4 µm, 150 x 2.0 mm) analytical column. An AB Sciex API 3000 mass spectrometer at unit resolution in the multiple reaction monitoring mode was used to monitor the transition of the protonated precursor ions m/z 472.9 and m/z 480.2 to the product ions m/z 431.0 and m/z 432.1 for clofazimine and the internal standard, respectively. The calibration curve fitted a quadratic (weighted by 1/concentration) regression over the range 0.00781 – 2.00 µg/mL. The accuracies (%Nom) were between 100.1% and 105.0%, with precision (%CV) less than 7.9% at the low, medium, and high-quality control concentrations during inter-batch validation.

Results

Table S1. Covariates relationship

	Clofazimine alone	Clofazimine with other QT prolonging drugs	p-value*
Age; median (2.5th-97.5th centiles) in years	2.7 (0.4 , 15.9)	12.3 (1.7 , 16.0)	0.0008
Living without HIV n=49 (91%)	41 (84)	8 (16)	0.052
Living with HIV n=5 (9%)	2 (40)	3 (60)	

Normal weight n=48 (89%)	39 (81)	9 (19)	0.590
Undernourished[a] n=6 (11%)	4 (67)	2 (33)	
Male n=25 (46%)	23 (92)	2 (8)	0.036
Female n=29 (54%)	20 (69)	9 (31)	

*p-value was calculated based on Wilcoxon-Mann-Whitney test for continuous variable and for categorical variable was based on Chi squared test or Fisher's exact test when more than 20% of the cells have expected frequencies < 5. [a] Obtained from weight for age z-score for children < 5 years [n = 36] and body mass index for age z-score for children ≥ 5 years [n = 28].

Table S2. Clofazimine PK and ECG information

**Variable**	**Value**
PK samples (N = 54)	
Total number of PK samples	370
Occasion 1 (n = 54)	214
Occasion 2 (n = 34)	136
Occasion 3 (n = 5)	20
ECG measures (N = 54) in children with PK data	
Total number of ECG measurements	1156
Occasion 1 (n = 54)	629
Occasion 2 (n = 39)	455
Occasion 3 (n = 6)	72

Occasion is a PK-day for the same patients

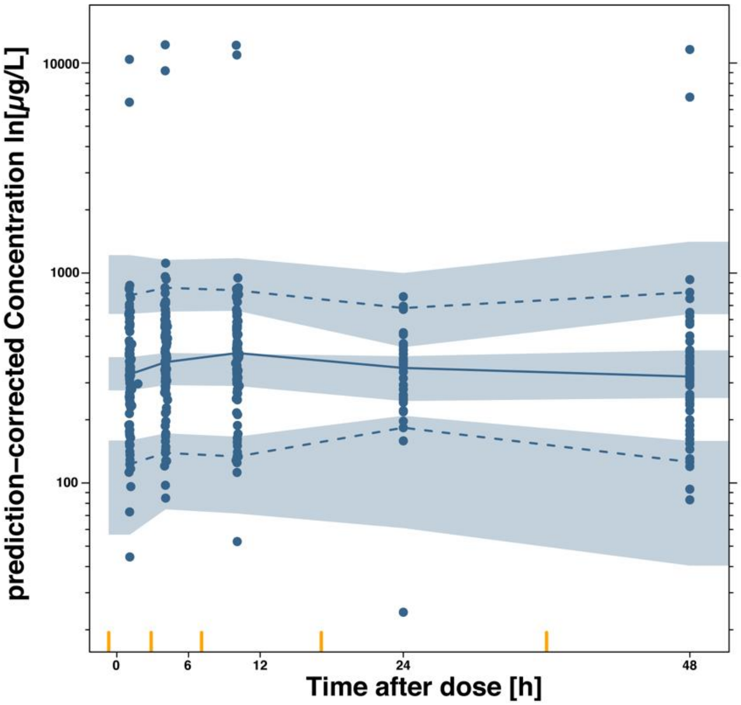
prediction-corrected Concentration ln[μg/L]
10000
1000
100
0
6
12
24
48
Time after dose [h]

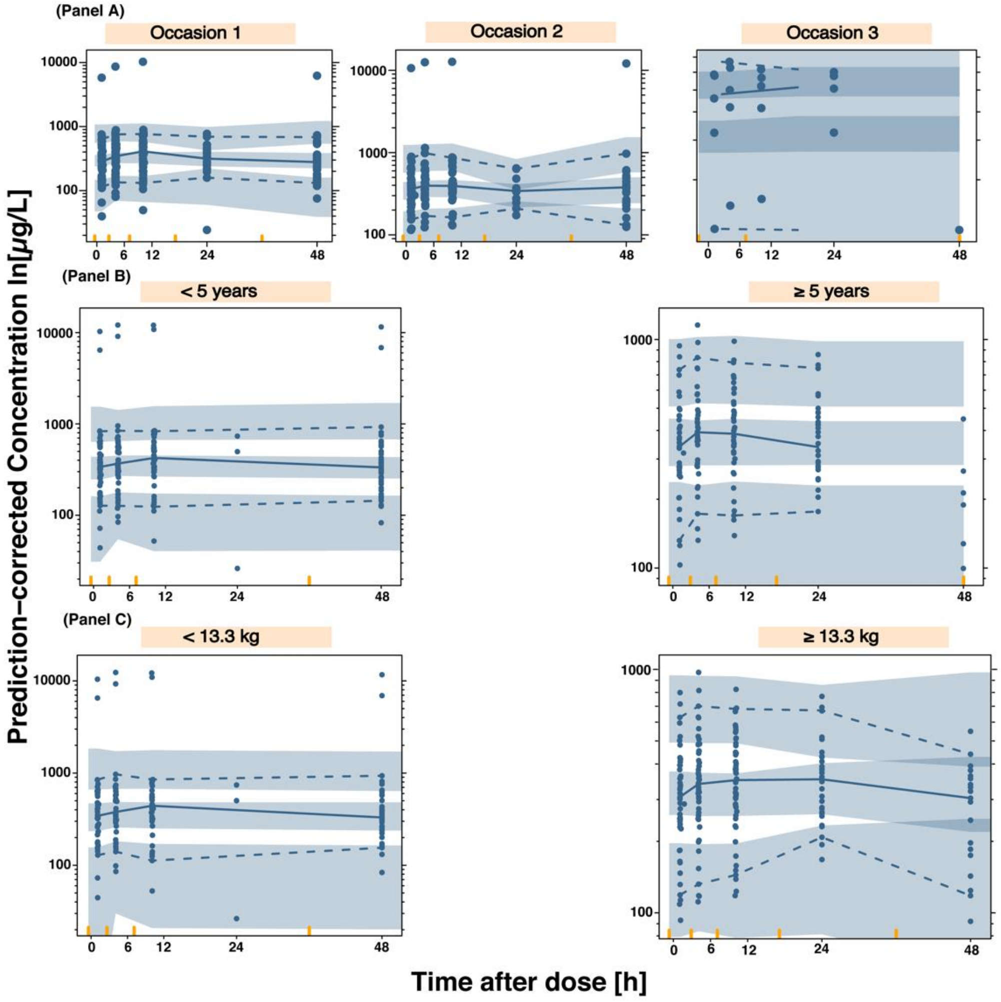
(Panel A)
Occasion 1
Occasion 2
Occasion 3
(Panel B)
< 5 years
≥ 5 years
(Panel C)
< 13.3 kg
≥ 13.3 kg
Prediction-corrected Concentration ln[μg/L]
10000
1000
100
0
6
12
24
48
Time after dose [h]

Figure S1. Prediction-corrected visual predictive check of the final model describing the plasma concentration of clofazimine over time after dose (top panel) in children with rifampicin-resistant tuberculosis. Panel A is predicted-corrected visual predictive check stratified by sampling occasions. Panel B is predicted-corrected visual predictive check stratified by age. Panel C is predicted-corrected visual predictive check stratified by weight, the cut-off for the weight is based on the median value of the study population. Closed circles represent the observed data points; solid and dashed lines represent the 50th, 5th, and 95th percentiles of the observed data, respectively; shaded areas correspond to the 95% confidence interval after 1,000 simulations for the corresponding percentile.

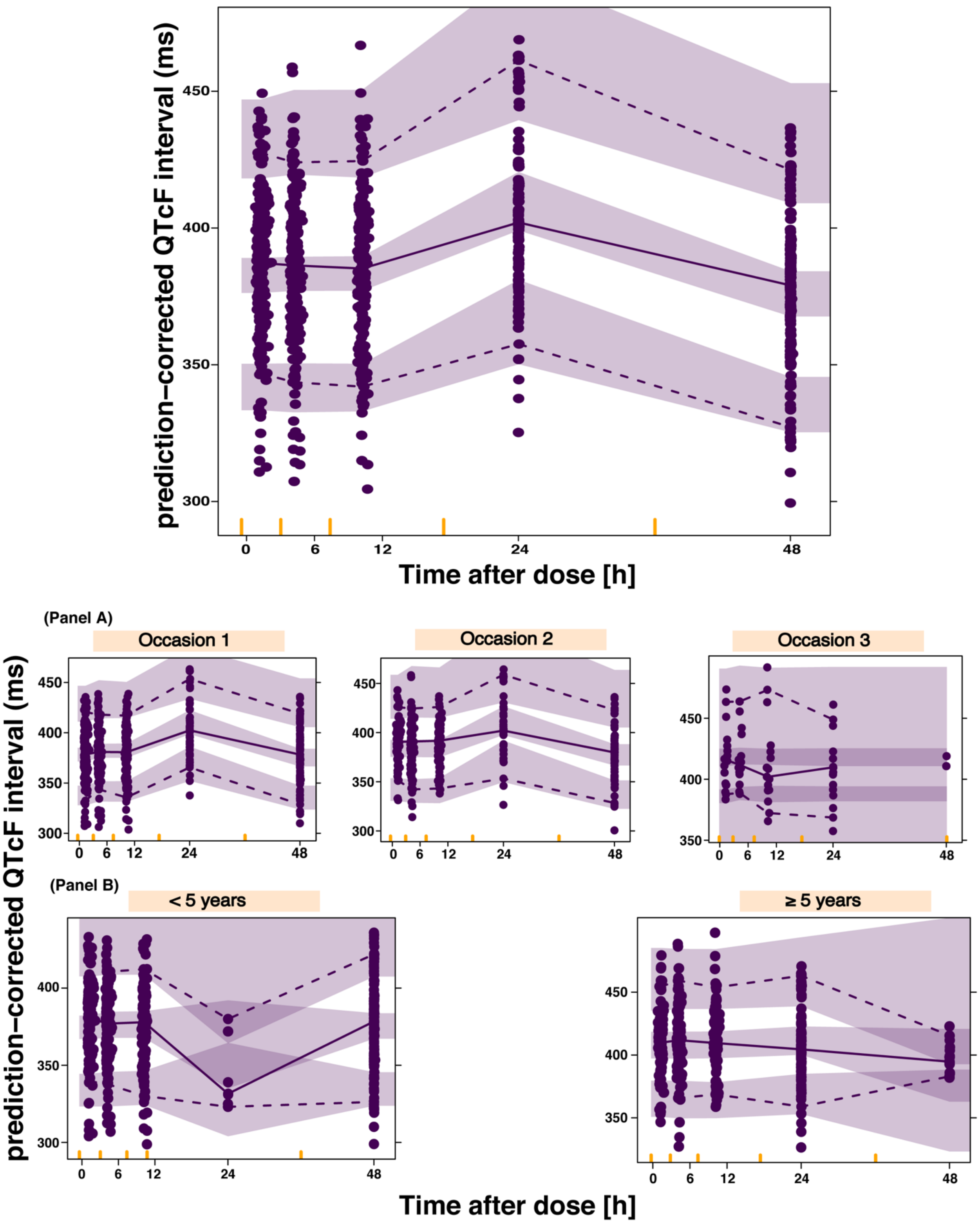

Figure S2. Prediction-corrected visual predictive check of the final model describing the QTcF interval versus time after dose (top panel). Panel A is predicted-corrected visual predictive

check stratified by sampling occasions. Panel B is predicted-corrected visual predictive check stratified by age. Closed circles are the observed data points; solid and dashed lines are the 50th, 5th, and 95th percentiles of the observed data; shaded areas are the simulated (n=1000) 95% confidence interval for the same percentile. QTcF = QT interval corrected by Fridericia formula.

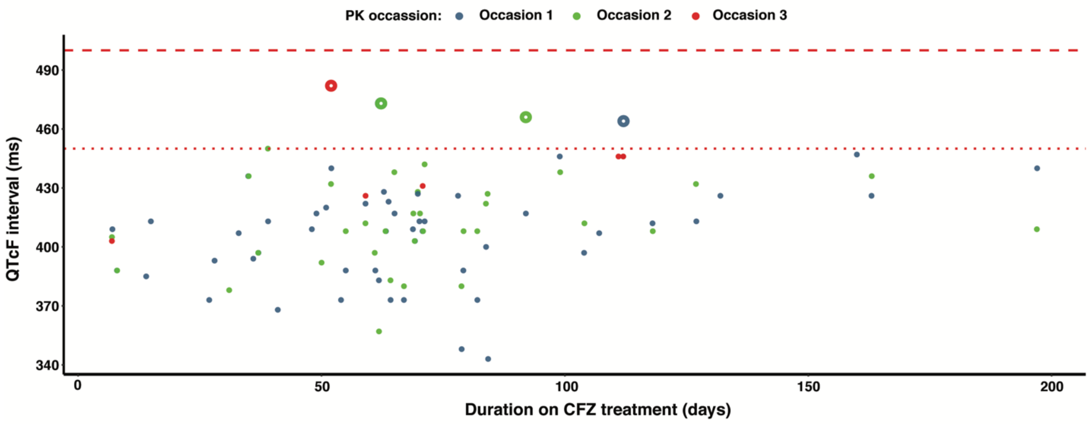

Figure S3. Maximum QTcF over treatment duration by PK occasion. Each solid circle represents a maximum QTcF. Solid circles are QTcF values less than or equal to 450 ms. Open circles are QTcF values greater than 450 ms. Doted red line represents a QTcF of 450 ms and dashed red line represents a QTcF of 500 ms (grade 3 adverse event). QTcF = QT interval corrected by Fridericia formula.
